# Encouraging Indonesians to Pray From Home During the COVID-19 Pandemic

**DOI:** 10.1017/XPS.2020.26

**Published:** 2020-07-15

**Authors:** Nicholas Kuipers, Saiful Mujani, Thomas Pepinsky

**Affiliations:** 1 Department of Political Science, University of California, Berkeley, CA, USA; 2 Department of Political Science, UIN Jakarta, South Tangerang, Indonesia; 3 Department of Government, Cornell University, Ithaca, NY, USA

**Keywords:** COVID-19, Islam, Indonesia, survey experiment

## Abstract

Despite the introduction of social restrictions designed to stem the spread of COVID-19, many Indonesians have continued to attend places of worship. This poses a major public health threat, as congregational prayer involves large numbers of worshippers gathering under conditions known to enable the spread of the virus. Using a nationally representative survey, we evaluated the efficacy of messages delivered from different authorities in encouraging Indonesians to worship at home. We find no consistent evidence that public health messages change Indonesians’ attitudes toward communal prayer or their willingness to forgo communal prayer during the COVID-19 pandemic. Importantly, however, looking at well-defined subpopulations – non-Muslims and supporters of the president – we find suggestive evidence that messages were effective in increasing the likelihood of individuals to indicate a willingness to forgo communal prayer in the forthcoming week. Our results suggest that public health officials should eschew blanket messaging strategies in favor of more targeted approaches.

## Introduction

On March 15, the Indonesian government called for mass social restrictions in response to the ongoing COVID-19 pandemic. Yet, 3 months later, Indonesians continue to attend social gatherings – especially religious events. In a national survey conducted in May 2020, for instance, 42% of respondents reported having attended a religious establishment in the previous week, meaning that an estimated 105 million Indonesians flaunted the advice of government officials. This poses a major public health threat, as congregational prayer involves large numbers of worshippers gathering under conditions known to enable the spread of the virus. One explanation for this behavior is that the cost of forgoing congregational prayer might appear to be high exceptionally high in Indonesia: 88% of the population adheres to Islam, which calls upon followers to pray five times a day (*sholat*), a practice that is generally observed in group settings (*jemaah*).^1^ Observational evidence partially supports this possibility. In the same survey, 44% of Indonesian Muslims reported having attended a place of worship in the last week, compared to just 19% of Indonesian Christians.

Another explanation, however, concerns the perceived authority of the Indonesian government in directing religious activities. Recently re-elected to a second term, President Joko Widodo (known universally as “Jokowi”) has faced challenges from hard-line Islamist elements in Indonesian society. These conflicts have tarnished Jokowi’s religious credentials in the eyes of many Indonesians.^2^ We believe that these broader political currents could affect the likelihood that Indonesians will comply with restrictions on places of prayer. If the perceived costs of forgoing congregational prayer are high, it might be the case that leaders or institutions with strong religious credentials could provide “cover” to individuals for whom the subjective utility of attendance is comparatively low, but who choose to attend devotional gatherings anyway.

This particular explanation builds on a large literature in political psychology which suggests that individuals’ attitudes and behaviors are influenced by elite cues (Zaller 1992). However, these effects are circumscribed by citizens’ evaluation of whether leaders are in-group members (Arriola and Grossman forthcoming; McCauley 2014). In the American context, where partisanship has become an entrenched cleavage, Republicans and Democrats tend to “follow the leader” in adopting positions held by co-partisan politicians (Broockman and Butler 2017; Lenz 2013). Worryingly, this tendency is amplified among highly politicized individuals (Druckman, Peterson, and Slothuus 2013; Guisinger and Saunders 2017). These findings have serious consequences in the context of the COVID-19 pandemic, with politicians issuing public health directives to their populations who are variously inclined to comply. A growing number of recent unpublished studies have found that compliance with stay-at-home directives in the USA is influenced by individuals co-partisanship with local authorities (Grossman, Kim, Rexer, and Thirumurthy. Unpublished manuscript, 2020; Alcott et al. Unpublished manuscript, 2020). In the field of public health communication, standard accounts have long focused on the health information sources in explaining the effectiveness of public health communication (McGuire 1989; Kreuter and McClure 2004).

Such effects are likely present in Indonesia, albeit in a different form. Party identification is not an important distinction in Indonesia.^3^ However, with the world’s largest population of Muslims, and a sizable minority population, religion and religiosity are salient cleavages in Indonesian politics – and politicians vying for elected office typically either signal or downplay their devotional credentials as a means of courting different constituencies (Aspinall, Dettman, and Warburton 2011; Fossati et al. 2020). The cleavage is not primarily one of religious *affiliation*, but rather one of religious *identity* among Indonesia’s Muslim population. One side of this cleavage emphasizes a more conservative interpretation of Islam and seeks to expand the visible role of Islam in public and political life. The other side of this cleavage – identified sometimes as nationalist or syncretic – acknowledges that most Indonesians are Muslims but celebrates Indonesia’s multireligious heritage. For this reason, the roughly 11% of Indonesians who are not Muslim also fall on this side of Indonesia’s identity cleavage.

The recent 2019 presidential election verified importance of this division, leading many observers to conclude that religious identity has become the country’s central political cleavage (Pepinsky 2019). President Jokowi, facing suspicions that he lacked proper Muslim credentials and was insufficiently attentive to Muslim concerns, named influential but nonpartisan cleric Ma’ruf Amin as his Vice President to shore up Muslim support, whereas the opposition ticket of Prabowo Subianto and Sandiaga Uno embraced the Islamic side of the cleavage. Religious signals, moreover, have been shown to affect voter attitudes and behaviors (Pepinsky, Liddle, and Mujani 2012). Consistent with findings on the role of elite cues from elsewhere across the world, recent work has found that Indonesian voters “follow the leader” along this religious dimension, adopting attitudes, and behaviors consistent with religiously inclined politicians (Kuipers, Nellis, and Weaver 2019).

Given these dynamics, and the ongoing COVID-19 pandemic, our overarching expectation was that the efficacy of messages discouraging congregational prayer in Indonesia would hinge on the receiver’s evaluation of sender’s authority. We evaluated the efficacy of different messages being used to encourage citizens to stay away from congregating in large numbers at religious establishments. Our experimental approach involved evaluating the efficacy of two genres of intervention: institutional appeals and personal appeals. Personal appeals are those made by (or in the name of) individual politicians, which may be viewed more favorably among respondents due to the perceived authority, trustworthiness, or charisma of the individual. Institutional appeals, by contrast, do not specify a particular individual, but instead evoke the authority of a broader institution (religious, government, or otherwise). We registered a series of hypotheses and parallel analyses to test these propositions. The review process was conducted prior to the realization of the data used to test our claims.

Using a phone survey of randomly sampled Indonesian voting-age citizens, we evaluated respondents’ responses to encouragements from two prominent political personalities: (1) an endorsement from President Joko Widodo and (2) an endorsement from Vice President Kyai Ma’ruf Amin. As discussed, the general public’s belief in the religiosity of Jokowi has been contested in recent years; meanwhile Ma’ruf Amin is a Muslim cleric with a reputation for religiosity. We compare these to the efficacy of encouragement messages from institutional sources, both of which vary according to perceived religiosity: (1) endorsements from two of Indonesia’s main religious organizations and (2) endorsements from the Indonesian Ministry of Health. All four messages derive from true factual statements made by these officials and institutions over the past few weeks discouraging citizens from congregating in prayer.

## Hypotheses

We registered three primary hypotheses (see Table 1). First, we expected that respondents in any experimental condition (T1, T2, T3, T4), when compared against respondents in a control condition (C), (a) would report stronger support for policies closing places of prayer and (b) would state a higher likelihood of avoiding religious establishments in the coming week. Second, we anticipated that respondents receiving messages from religiously affiliated institutional and personal appeals (T1, T4), compared against respondents who receive messages from more secular institutions and personalities (T2, T3), (a) would report stronger support for policies closing places of prayer and (b) would state a higher likelihood of avoiding religious establishments in the coming week. Finally, third, we hypothesized that respondents receiving personal appeals (T3, T4), compared against those receiving institutional appeals (T1, T2), (a) would report stronger support for policies closing places of prayer and (b) would state a higher likelihood of avoiding religious establishments in the coming week.^4^

**Table 1:** Primary Hypotheses

Hypothesis	Analysis plan
H1: Any institutional or personal encouragement increases compliance with religious restrictions	For both dependent variables, estimate Model (1) and test the null hypothesis that *β*_1_ = 0. If *β*_1_ > 0, we reject the null that *β*_1_ = 0
H2: Any institutional or personal encouragement from a religious authority increases compliance with religious restrictions more than an institutional or personal encouragement from a secular authority	For both dependent variables, estimate Model (1) and test the null hypothesis that *β*_1_ = 0. If *β*_1_ > 0, we reject the null that *β*_1_ = 0
H3: Any personal encouragement increases compliance with religious restrictions more than an encouragement from a institutional authority	For both dependent variables, estimate Model (1) and test the null hypothesis that *β*_1_ = 0. If *β*_1_ > 0, we reject the null that *β*_1_ = 0

Our approach conceptualizes the decision to forgo congregational prayer as a product of individuals weighing subjective costs and benefits. To bolster this interpretation, we registered two further “secondary” hypotheses that probe for heterogeneity in the main effects (see Table 2). First, one of Islam’s five pillars, *salah*, calls for daily prayer, which is generally observed in group settings.^5^ We therefore expected that the costs of forgoing congregational prayer would be higher for Muslim respondents than for Christian respondents. As a result, we hypothesized that the efficacy of the messages would larger for Christians than Muslims. Second, we also expected that our messages would be most effective in swaying the attitudes and behaviors of weakly pious individuals, for whom the subjective cost of forgoing congregational prayer is likely lower. Out of concerns of statistical power, we evaluated these secondary hypotheses in a manner similar to H1.

**Table 2:** Secondary Hypotheses

Hypothesis	Analysis plan
H4: Any institutional or personal encouragement increases compliance with religious restrictions more for Christians than Muslims	For both dependent variables, estimate Model (2) and test the null hypothesis that *β*_3_ = 0. If *β*_3_ < 0, we reject the null that *β*_3_ = 0
H5: Any institutional or personal encouragement increases compliance with religious restrictions more for the weakly pious than for the strongly pious	For both dependent variables, estimate Model (3) and test the null hypothesis that *β*_3_ = 0. If *β*_3_ < 0, we reject the null that *β*_3_ = 0
H6: Any institutional or personal encouragement increases compliance with religious restrictions more for the *a priori* noncompliant than for the already-compliant	For both dependent variables, estimate Model (4) and test the null hypothesis that *β*_3_ = 0. If < 0, we reject the null that *β*_3_ = 0
H7: Any institutional or personal encouragement increases compliance with religious restrictions more for Jokowi supporters than for Jokowi opponents	For both dependent variables, estimate Model (5) and test the null hypothesis that *β*_3_ = 0. If *β*_3_ < 0, we reject the null that *β*_3_ = 0

We are centrally interested in which messages are effective in moving the attitudes and behaviors of *a priori* noncompliers – i.e., individuals who reported attending a place of worship in the preceding week, despite recommendations to the contrary. Attitudinal support for restrictions on places of worship is likely already high among behaviorally compliant respondents. It is also likely that respondents who reported not having attended a place of worship in the previous week will likely not attend a place of worship in the forthcoming week. This suggests that our interventions might run up against “ceiling effects” among the already compliant respondents. When compared against respondents who report not having attended a mosque in the previous week, we therefore hypothesized that our interventions would have larger effects among these noncompliers.

Finally, we also considered the possibility that the effects of these messages vary by respondents’ political orientation to the current government. Specifically, supporters of the current Jokowi administration may be more responsive official messages than those who oppose the current administration. Because Jokowi’s Vice President Ma’ruf Amin has strong ties to Indonesian Ulema Council, this sensitivity should also apply to messages that are not explicitly political in origin.

## Methods

### Design

In line with the hypotheses outlined above, we evaluated the comparative efficacy of four different encouragements. Specifically, we used a procedure of simple randomization to assign respondents to hear one of the five following messages:

- **Control (C):** Respondent received the following message: “Around 2–3 months ago, some figures asked all citizens to conduct religious ceremonies and prayer at home, and not gather in congregational worship at mosques, churches, or temples. These actions will help stop the spread of COVID-19.”
- **Religious institutional (T1):** Respondent received the following message: “Around 2–3 months ago, the Indonesian Ulema Council (*Majelis Ulama Indonesia*, MUI) and the Christian Association of Churches asked all citizens to conduct religious ceremonies and prayer at home, and not gather in congregational worship at mosques, churches, or temples. These actions will help stop the spread of COVID-19.”
- **Nonreligious institutional (T2):** Respondent received the following message: “Around 2–3 months ago, the Indonesian Ministry of Health asked all citizens to conduct religious ceremonies and prayer at home, and not gather in congregational worship at mosques, churches, or temples. These actions will help stop the spread of COVID-19.”
- **Nonreligious personal (T3):** Respondent received the following message: “Around 2–3 months ago, President Joko Widodo asked all citizens to conduct religious ceremonies and prayer at home, and not gather in congregational worship at mosques, churches, or temples. These actions will help stop the spread of COVID-19.”
- **Religious personal (T4):** Respondent received the following message: “Around 2–3 months ago, Vice President Kyai Ma’ruf Amin asked all citizens to conduct religious ceremonies and prayer at home, and not gather in congregational worship at mosques, churches, or temples. These actions will help stop the spread of COVID-19.”

We measured the efficacy of these messages according to two outcomes: an attitudinal measure and a behavioral measure, which we asked respondents directly after hearing the encouragement. The attitudinal measure asked respondents “to what extent do you support or not support restrictions on congregational prayer?” Respondents chose from four options: strongly support, somewhat support, somewhat not support, and strongly not support. Our behavioral outcome asked respondents “do you plan to attend a mosque, church, or temple in the coming week?” Respondents chose from either “yes” or “no.”^6^

To evaluate our secondary hypotheses, we attempted to collect additional data from respondents concerning their religion, religiosity, compliance, and partisanship. On the first count, we asked respondents to state their religion at the end of the survey (Indonesians are legally required to profess one of six officially recognized religions). On the second count, we had also intended to collect data from previous surveys gauging religiosity that asked respondents “In your own opinion, how devout would you say you are with respect to your religion?” This question has been asked on previous surveys, from which the respondents were selected meaning that we could link these responses to the current survey. However, the survey firm was unable to link our survey with the earlier rounds of data, meaning we were unable to test the relevant hypotheses. As a measure of compliance, before the experimental module, we asked respondents whether they had attended a place of worship in the week prior. Finally, fourth, as a measure of partisanship, we asked respondents who they voted for in the recent presidential election.^7^ Using these pre-intervention data, we intended to categorize respondents as “Muslim” or “non-Muslim,” “pious” or “not pious,” “complier” or “noncomplier,” and “Jokowi supporter” or “nonsupporter.”

### Sampling plan and power analyses

We conducted a telephone survey of 1,985 voting-age Indonesian citizens between June 18 and 20, 2020.^8^ The sampling procedure was conducted as follows. Respondents’ phone numbers were randomly drawn from a database privately maintained by the survey firm. The universe of potential phone numbers (i.e., the database) was defined as individuals (1) who, in the last 3 years, were part of a face-to-face survey conducted by the survey firm and (2) had a telephone at the time that survey was conducted.^9^ Importantly for the external validity of our sample, the initial construction of this universe of respondents was randomly selected to constitute a representative sample of Indonesia’s voting age population. As a validation check, the survey firm conducted telephone surveys according to this procedure during the most recent Indonesian presidential election, finding the results to be accurate within half a percentage point of the final results. The results of our pre-analysis power calculations indicated that the proposed sample size was sufficient to detect substantively meaningful effects at least 80% of the time (see Figure 1 in the Supplementary Appendix).

**Figure 1:**
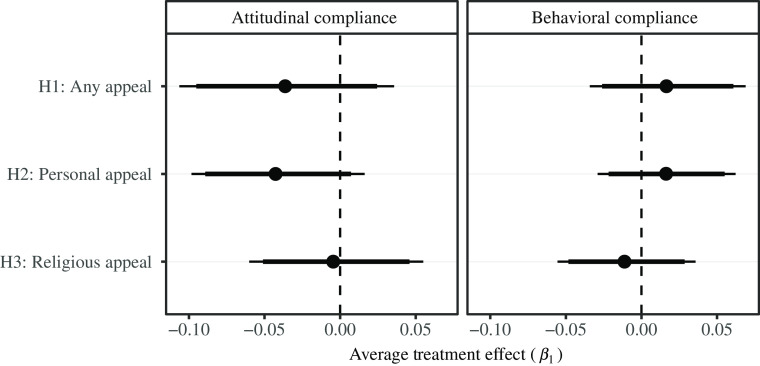
Primary Analysis, Hypotheses 1a–3b.

*Note:* Beta coefficients estimated from equation (1). Conventional standard errors calculated at the individual level. 95% and 90% confidence intervals are included.

### Analysis plan

All outcomes were scaled such that higher values denote greater support for social distancing restrictions. Our attitudinal measure was constructed on a four-point Likert scale and our behavioral measure was constructed as a binary indicator. We leave this coding scheme undisturbed in our main analyses. Our analysis adopted a difference-in-means framework, implemented using ordinary least squares regression. We calculated heteroskedastic-consistent standard errors. For our three primary hypotheses, we isolated the effect of different messages on the outcomes of interest using the following baseline specification:
(1)
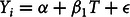

where *Y* is a stand-in for the outcome of interest for respondent *i*. *T* is a treatment assignment vector that varies according to the hypothesis being tested. For hypotheses H1a and H1b, *T* takes a “1” if respondent *i* was assigned to either T1, T2, T3, or T4 and a “0” if respondent *i* was assigned to receive C. For hypotheses H2a and H2b, *T* takes a “1” if respondent *i* was assigned to either T1 or T4 and a “0” if respondent *i* was assigned to receive either T2 or T3. Finally, for hypotheses H3a and H3b, *T* takes a “1” if respondent *i* was assigned to either T3 or T4 and a “0” if respondent *i* was assigned to receive either T1 or T2. All other instances are assigned missing values. Here, *β*_1_ is the parameter of interest, which we interpret as the average treatment effect of receiving a given message on the outcomes of interest.

For our secondary and tertiary hypotheses, we probed for the presence of an interaction effect drawing on additional variables available from the earlier surveys. Specifically, we estimated three models:
(2)
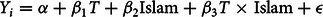

(3)
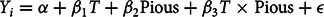

(4)
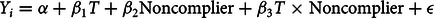

(5)
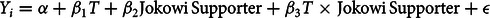

where again, *Y* is a stand-in for the outcome of interest for respondent *i* and *T* is a treatment assignment vector. In this case, recall we are probing for heterogeneity in effects under hypotheses H1a and H1b only, such that *T* takes a “1” if respondent *i* was assigned to either T1, T2, T3, or T4 and a “0” if respondent *i* was assigned to receive C. Islam is a vector that takes a “1” if respondent *i* is a Muslim and a “0”otherwise. Pious is a vector that takes a “1” if respondent *i* self reported that they were either “somewhat devout” or “strongly devout” on the pre-intervention survey. Noncomplier is a vector that takes a “1” if respondent *i* reported having attended a place of worship in the past week. JokowiSupporter is a vector that takes a “1” if respondent *i* reported having voted for Jokowi in 2019.

We deviated from our pre-analysis plan at two important junctures in the implementation of our research protocol and the subsequent analysis. First, we had committed to measure our “behavioral” outcome on a four-point Likert scale. Our initial protocol, however, stipulated a binary measure of intent to attend a place of worship in the forthcoming week. This change was not communicated to the survey firm, and respondents were instead offered the binary choice variable. While unfortunate, we believe this is a tolerable error since binary outcome variables are generally more conservative. Second, we had initially proposed to use earlier measures of piety to evaluate H6. This expectation was driven by a miscommunication with the survey firm, which is in fact unable to link the results of our survey to earlier data.

## Results

To begin, how does hearing *any* encouragement to pray from home affect the likelihood of respondents to support the restrictions on places of prayer? The results indicate that respondents who received any encouragement (T1, T2, T3, T4) were no more likely than respondents who received no encouragement (C) to state support for restrictions on places of prayer (*β*_1_ = −0.035, p = 0.32). They were also no more likely to indicate that they would change their behavior in the forthcoming week (*β*_1_ = 0.017, p = 0.51). We also hypothesized that receiving a personal appeal would be more effective than receiving an institutional appeal. However, we fail to detect evidence that respondents who received an encouragement to pray from home from a personality, compared against those who received a message from an institution, were any more likely to support restrictions on places of prayer (*β*_1_ = −0.041, p = 0.16), nor were they any more likely to indicate a willingness to change their behavior (*β*_1_ = 0.017, p = 0.47). Finally, third, we further hypothesized that individuals who received encouragements to pray from home from individuals and institutions with strong religious credentials, as opposed to individuals and institutions perceived to be more secular, would be more willing to adopt attitudes and behaviors consistent with the recommendations. Again, however, we do not observe that religiously tinged appeals were any more effective at either encouraging individuals to adopt attitudes supportive of restrictions on places of prayer (*β*_1_ = −0.00, p = 0.93) or increasing individuals’ stated likelihood of complying with restrictions in the forthcoming week (*β*_1_ = −0.01, p = 0.67).

**Figure 2:**
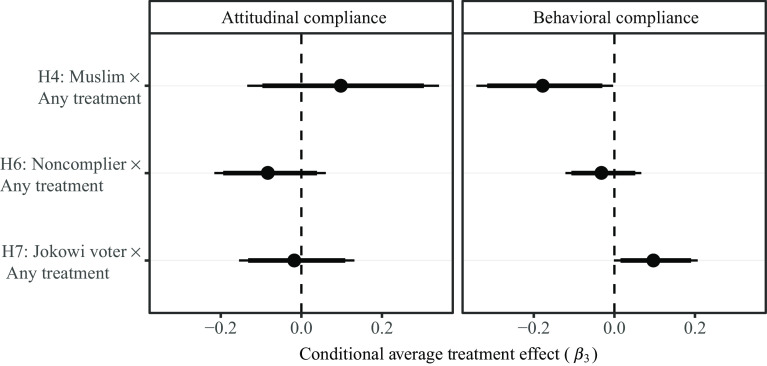
Primary Analysis, Hypotheses 4a–7b.

*Notes:* Beta coefficients estimated from equations (2)–(5). Conventional standard errors calculated at the individual level. 95% and 90% confidence intervals are included.

Although we detect no treatment effects that are statistically distinguishable from zero in our main analysis, it might be the case that encouragements were effective for specific subpopulations, as we hypothesized in H4a–H7b. Recall that for logistical reasons relating to the rollout of the survey, we were unable to test H5a and H5b. We present our analysis in Figure 2. First, were encouragements less effective at changing attitudes and behaviors among Muslims, compared against non-Muslims? Our results indicate that encouragements were no more effective at changing the attitudes of non-Muslims, compared against Muslims. However, we find that any encouragement to pray from home was more effective at increasing the likelihood of non-Muslims to indicate that they would not attend a place of worship in the forthcoming week, when compared against Muslims (*β*_3_ = −0.17, p = 0.046).

We also hypothesized that any encouragement to pray from home might be more effective at changing attitudes and behaviors of individuals who voted for Jokowi in the recent 2019 presidential election, compared against those who did not. Again, we find no evidence that encouragements to pray from home were more effective at changing the attitudes of Joko Widodo supports, compared against nonsupporters. However, upon hearing any encouragement, Jokowi voters were somewhat more likely to indicate a willingness to pray from home in the forthcoming week, compared against nonsupporters (*β*_3_ = 0.10, p = 0.052).^10^

Are our null results a function of limited power to detect small effects? Our preregistered power analysis assumed the smallest effect size to be 0.05 and a sample size of 1,600 (see Figure 1, Supplementary Appendix). However, our estimates produced smaller effect sizes (e.g., *β*_3_ = 0.017 for H1a). We had anticipated that – in the event of any effects – we would observe larger swings in attitudinal and behavioral compliance. Given our *actual* sample size of 1,985 respondents, though, we have sufficient power to detect effects of as small as 0.03 for H1–H3. This is enough to rule out insufficient power as an explanation for null results for H2 and H3. While it is possible that the true effect sizes that we are trying to estimate for the other hypotheses are smaller still, this is reassuring evidence that our null results for these main analyses are not a function of low statistical power.

## Discussion

Our experimental analysis has uncovered no consistent evidence that public health messaging changes Indonesians’ attitudes toward restrictions on communal prayer or their willingness to forgo communal prayer during the COVID-19 pandemic. These findings are discouraging for public health communication, for such low-cost approaches as simple telephone reminders do not appear to be effective at changing attitudes or behaviors. Future research may be able to establish if a more direct and sustained – but perhaps more costly – messaging campaign is more effective. It may also be that focusing on less politically fraught social activities (e.g., shopping, going to work, or visiting family members) would have different effects. Alternatively, in the present sociopolitical environment of highly salient discussions of the health effects of COVID-19, additional information from public health messaging may be unable to change mass preferences or behaviors.

We also highlight a secondary finding: we find no evidence that the source of the public health message affects attitudes or behaviors either. Messages from religiously affiliated authorities are no more effective than are appeals from more secular state authorities, and personal appeals are no more effective than institutional appeals. These results do not allow us to conclude that the sources of public health information do not matter for changing attitudes and behavior, but they suggest that in the context of public health campaigns, mass publics may be less sensitive to the sources of public health information than often believed. These findings are consistent with several other null results from endorsement experiments in other national contexts (Bhanot and Hopkins 2020; Gadarian, Goodman, and Pepinsky 2020) during the COVID-19 pandemic.

We do find suggestive evidence that public health messages were effective for well-defined subpopulations: public health messages were less effective among Muslims, and more effective among supporters of the sitting president. Interpreted through the literature on public health communication (McGuire 1989; Kreuter and McClure 2004), these results suggest that respondent characteristics rather than sender characteristics are what determine the effectiveness of public health messages. Further studies of public health communication may profitably investigate communication strategies that target communities who are less responsive to public health messaging – in the contemporary Indonesian case, Muslims and opponents of Jokowi.

## Footnotes

1 According to a March 2020 survey, before social restrictions were put in place, 77% of Indonesian Muslims reported praying five times a day.

2 In an April 2019 survey, for instance, 19% of Indonesians agreed that Jokowi could be characterized as “anti-Islam,” even though he is himself Muslim.

3 In an April 2019 survey, only 12.2% of Indonesians stated that they felt close to any particular party.

4 It is possible that the effect of religiously affiliated (versus secular) messages or personally affiliated (versus institutional) messages could vary across the specific treatments. We therefore also estimated the effect of each individual treatment (T1, T2, T3, and T4) as measured against the control condition (C). Out of space considerations, we do not enumerate these hypotheses here, but instead list them in the Supplementary Appendix.

5 According to a March 2020 survey, 77% of Indonesian Muslims reported praying five times a day.

6 In our pre-analysis plan, we had registered asking respondents “How likely are you to attend a mosque, church, or temple in the coming week?” Respondents were offered a four-point scale ranging from “very likely” to “not at all likely.” Due to a miscommunication with the survey firm, our behavioral measure was kept as a binary outcome.

7 There is good reason to believe respondents were honest: in our unweighted sample, 57.1% of respondents reported voting for Jokowi, approximately 1.5 percentage points off the true value of 55.5%.

8 We had initially proposed a sample size of 1,600.

9 The cell phone penetration rate in Indonesia is high: in a face-to-face survey conducted in early March by the survey firm we use, 90.4% of respondents offered a cell phone number.

10 One concern here might be with ceiling effects. However, there was significant variation in *a priori* levels of compliance among Jokowi voters, suggesting this is not an issue: 43% of Jokowi supporters, and 34% of non-Jokowi supporters, did not attend a place of worship in the previous week. Similar variation holds for non-Muslims (64.5%) and Muslims (36.2%).

## Supplementary material

For supplementary material accompanying this paper visit https://doi.org/10.1017/XPS.2020.26. click here to view supplementary material
