# Supplementary material for: Encouraging Indonesians to Pray From Home During the COVID-19 Pandemic
Source: Journal of Experimental Political Science. 2020 Jul 15:1–12. doi: 10.1017/XPS.2020.26 (PMC7417989; doi:10.1017/XPS.2020.26)
Supplement: Supplementary file 1 [file S2052263020000263sup001.pdf]

Supplementary Appendix

A Reporting standards **2**
- A.1 Hypotheses . . . 2
- A.2 Subjects and context . . . 2
  - Eligibility and exclusion criteria. . . . 2
  - Recruitment. . . . 2
  - Survey dates. . . . 2
  - Settings of data collection. . . . 2
  - Response rate. . . . 2
- A.3 Allocation method . . . 2
  - Randomization procedure. . . . 2
  - Evidence of randomization. . . . 2
- A.4 Treatments . . . 3
- A.5 Results . . . 3
  - Outcome measures and covariates. . . . 3
  - CONSORT Participant Flow . . . 3
- A.6 Statistical analysis . . . 3

B Design tables **4**
- B.1 Primary hypotheses . . . 4
- B.2 Secondary hypotheses . . . 5

C Power Analyses **6**

D Individual hypotheses and analyses **8**

A Reporting standards

A.1 Hypotheses

We enumerate all primary and secondary hypotheses to be tested in Table 1 and Table 2 of the main text. See above. We additionally test the efficacy of our individual interventions, which we enumerate in the appendix. See below.

A.2 Subjects and context

Eligibility and exclusion criteria. Respondents were selected as a representative sample of voting-age Indonesians (e.g., 17 years and older). Respondents also had to possess a cell phone or landline phone, which was the method by which we conducted our survey.

Recruitment. The survey was conducted by Saiful Mujani Research and Consulting, a private survey firm located in Jakarta, Indonesia. They identified respondents for phone surveys as past respondents in their face-to-face surveys conducted pre-pandemic. Using this database of past respondents (who also possessed cell phone numbers at the time of initial interview) they randomly selected respondents for our survey).

Survey dates. June 18-20, 2020.

Settings of data collection. Enumerators were located in Jakarta during the phone call solicitations and surveys. Respondents were all across Indonesia.

Response rate. 90.2%. The survey firm had oversampled from our initial request and sampled 2,200 phone numbers, making contact and obtaining survey responses from 1985.

A.3 Allocation method

Randomization procedure. 2,200 potential phone numbers were randomly selected for interviews by the survey firm. After this step, each phone number was assigned an arbitrary "respondent ID" number. For each of the five treatment assignments, 440 respondent ID numbers were selected by a procedure of simple random assignment without replacement.

Evidence of randomization. See below: Figure 1. Looking at the demographic characteristics of respondents assigned to any treatment group—as opposed to those assigned to the control condition—we observe no statistically significant differences.

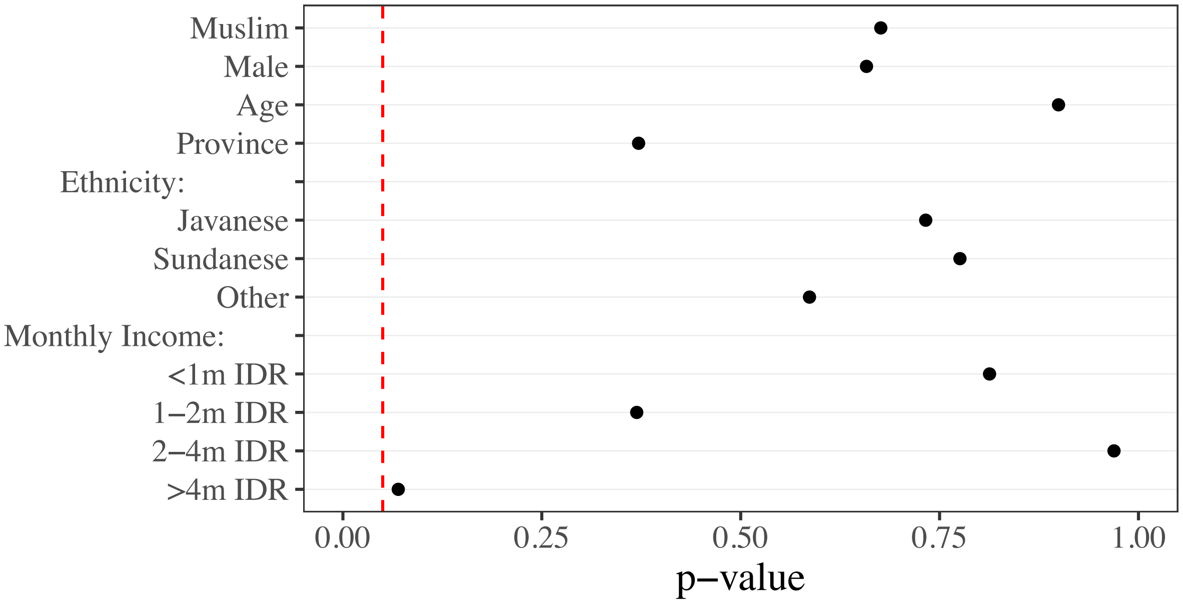

Figure 1—Balance tests on demographics. p-values from difference-means-tests implemented using ordinary least squares, in which we regress our "any treatment" indicator variable on the demographic variables presented above.

A.4 Treatments

See the main text of the paper.

A.5 Results

Outcome measures and covariates. See the main text of the paper for a description of the outcome measures. We do not use any covariate adjustments in our estimation.

CONSORT Participant Flow

- Number of subjects initially assessed for eligibility for the study: 2,200
- Exclusions prior to random assignment: 0
- Number of subjects initially assigned: 2,200 (440 for each condition)
- The proportion of each group that received its allocated intervention: 90.2% overall (1,985/2,200). Failure to contact on the respondents not receiving their intervention.
  - C: 86.1% (379/440)
  - T1: 94.3% (415/440)
  - T2: 96.3% (424/440)
  - T3: 88.4% (389/440)
  - T4: 85.90% (378/440)
- The number of subjects in each group that are included in the statistical analysis: 1,985

A.6 Statistical analysis

See main text of the paper for description. We do not use any weighting procedure.

B Design tables

B.1 Primary hypotheses

Table 1: Design Table—Primary Hypotheses

Question	Hypothesis	Sampling plan	Analysis plan	Interpretation
Q1: Do encouragements improve compliance with religious restrictions?	H1: any institutional or personal encouragement increases compliance with religious restrictions	See Figure 1a.	For both dependent variables, estimate Model (1) and test the null hypothesis that $\beta_1 = 0$	If $\beta_1 > 0$, and we reject the null that $\beta_1 = 0$, we have evidence of encouragements improving compliance. If we fail to reject the null, we have no evidence of improved compliance.
Q2: Do encouragements from religious authorities improve compliance with religious restrictions more than encouragements from secular authorities?	H2: any institutional or personal encouragement from a religious authority increases compliance with religious restrictions more than an institutional or personal encouragement from a secular authority	See Figure 1b.	For both dependent variables, estimate Model (1) and test the null hypothesis that $\beta_1 = 0$	If $\beta_1 > 0$, and we reject the null that $\beta_1 = 0$, we have evidence of religious encouragements improving compliance from than secular encouragements. If we fail to reject the null, we have no evidence of improved compliance from religious encouragements.
Q3: Do encouragements from institutional authorities improve compliance with religious restrictions more than encouragements from personal authorities?	H3: any personal encouragement increases compliance with religious restrictions more than an encouragement from a institutional authority	See Figure 1c.	For both dependent variables, estimate Model (1) and test the null hypothesis that $\beta_1 = 0$	If $\beta_1 > 0$, and we reject the null that $\beta_1 = 0$, we have evidence of personal encouragements improving compliance more than institutional encouragements. If we fail to reject the null, we have no evidence of improved compliance from personal encouragements.

B.2 Secondary hypotheses

Table 2: Design Table—Secondary Hypotheses

Question	Hypothesis	Sampling plan	Analysis plan	Interpretation
Q4: Do encouragements improve compliance with religious restrictions more for Christians than Muslims?	H4: any institutional or personal encouragement increases compliance with religious restrictions more for Christians than Muslims	See Figure 1d.	For both dependent variables, estimate Model (2) and test the null hypothesis that $\beta_3 = 0$	If $\beta_3 < 0$, and we reject the null that $\beta_1 = 0$, we have evidence of encouragements improving compliance more for Christians than Muslims. If we fail to reject the null, we have no evidence of improved compliance.
Q6: Do encouragements improve compliance with religious restrictions more for the *a priori*-noncompliant than the already-compliant?	H6: any institutional or personal encouragement increases compliance with religious restrictions more for the *a priori*-noncompliant than for the already-compliant.	See Figure 1f.	For both dependent variables, estimate Model (4) and test the null hypothesis that $\beta_3 = 0$	If $\beta_3 < 0$, and we reject the null that $\beta_1 = 0$, we have evidence of encouragements improving compliance more for the *a priori*-noncompliant than the already-compliant. If we fail to reject the null, we have no evidence of improved compliance.
Q7: Do encouragements improve compliance with religious restrictions more for respondents who support the Jokowi administration than those who oppose it?	H7: any institutional or personal encouragement increases compliance with religious restrictions more for Jokowi supporters than for Jokowi opponents.	See Figure 1g.	For both dependent variables, estimate Model (5) and test the null hypothesis that $\beta_3 = 0$	If $\beta_3 < 0$, and we reject the null that $\beta_1 = 0$, we have evidence of encouragements improving compliance more for Jokowi supporters than for Jokowi opponents. If we fail to reject the null, we have no evidence of improved compliance.

C Power Analyses

We conduct a series of power calculations to evaluate whether our proposed sample size is sufficient to detect statistically significant effects.[1] We conducted simulations assuming the same standard error across treatment and control means ($\sigma = 0.2$), at a 95% significance level, and assuming different effect sizes—all of which are in the range of reported treatment effects in similar endorsement experiments.[2] The results of our power simulations are reported in Figure 1. We include the vertical red line to indicate our proposed sample size for each test.

At our target sample size of 1600 respondents and target power of 95%, in testing H1a and H1b, we have sufficient power to detect treatment effects that are much smaller than 0.05 (corresponding to difference between treatment and controls of 5 percentage points), which would be among the small effects found in the literature. We likewise have sufficient power to detect a 5 percentage point difference in H2 and H3. For H4a-b and H5a-b, the effective decrease in sample size lowers our minimum detectable effect size. We estimate our minimum detectable effect size, at a target power of 95%, to be 9 percentage points for H4a and H4b, and 14 percentage points for H5a and H5b. We will not reject the null hypothesis of no interaction effects if our estimated interaction effects are smaller than these critical values. Finally, for H6a-b, we similarly estimate a minimum detectable effect size to be 9 percentage points at a target power of 95%.

1 Code for these analyses can be found in the Kuipers, Mujani and Pepinsky (2020) replication file.

2 For example, Pepinsky, Liddle and Mujani (2012) report an effect of approximately 0.09 in an analysis of an Islamist prime under conditions of policy uncertainty in Indonesia.

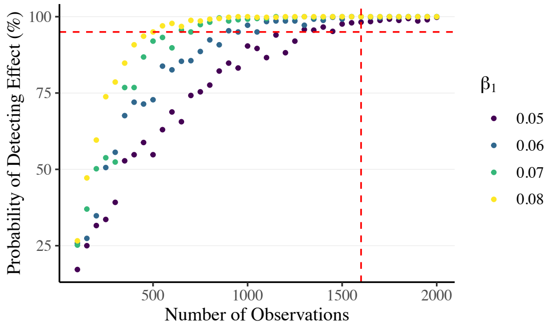

(a) H1 Power Test (β_1)

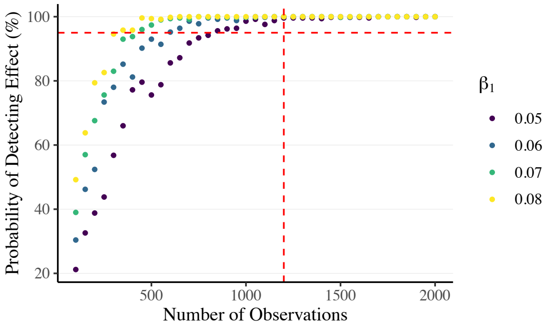

(b) H2 Power Test (β_1)

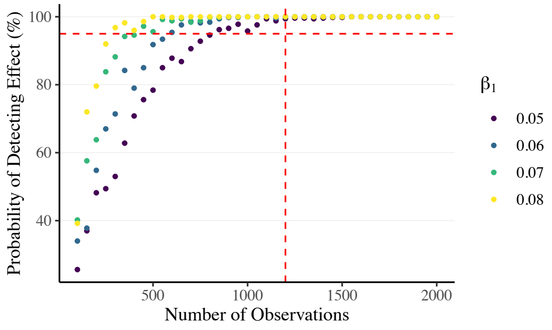

(c) H3 Power Test (β_1)

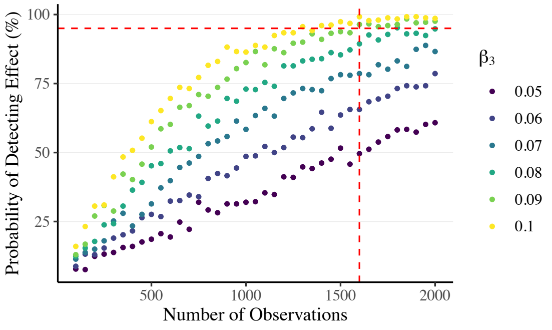

(d) H4 Power Test (β_3)

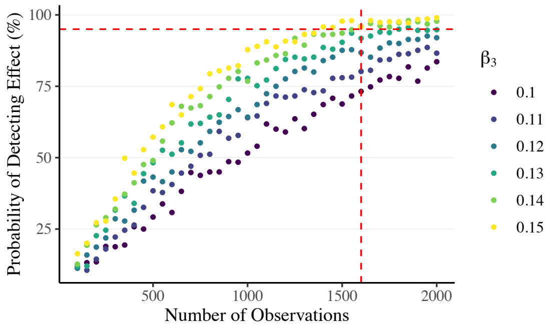

(e) H5 Power Test (β_3)

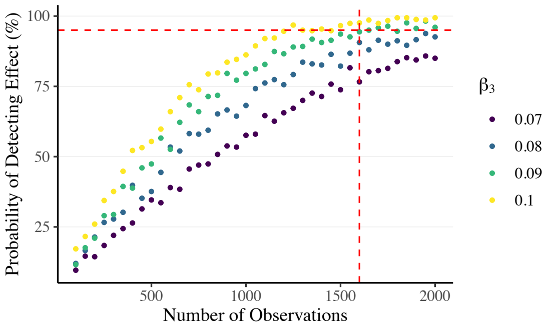

(f) H6 Power Test (β_3)

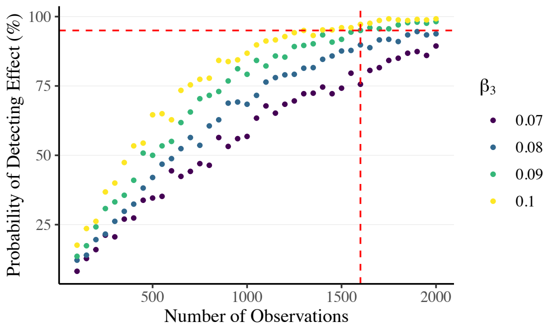

(g) H7 Power Test (β_3)

Figure 2—Power Calculations. Power tests conducted using 500 data simulations, over sample sizes ranging between 100 and 2,000 at intervals of 50 respondents. Horizontal lines indicate the threshold for detecting hypothesized effect sizes at least 95% of the time. Vertical lines indicate the proposed sample sizes for each test.

D Individual hypotheses and analyses

As discussed in the main text, we will also evaluate the efficacy of each individual message as measured against a control condition. Consistent with H1a and H1b, which expects that any nudge will have an effect, we hypothesize that all of our endorsements will increase both respondents' attitudinal support for praying at home and behavioral measures of their intent to do so.

- H8a: $E[Y_a|T1] > E[Y_a|C]$
- H8b: $E[Y_b|T1] > E[Y_b|C]$
- H9a: $E[Y_a|T2] > E[Y_a|C]$
- H9b: $E[Y_b|T2] > E[Y_b|C]$
- H10a: $E[Y_a|T3] > E[Y_a|C]$
- H10b: $E[Y_b|T3] > E[Y_b|C]$
- H11a: $E[Y_a|T4] > E[Y_a|C]$
- H11b: $E[Y_b|T4] > E[Y_b|C]$

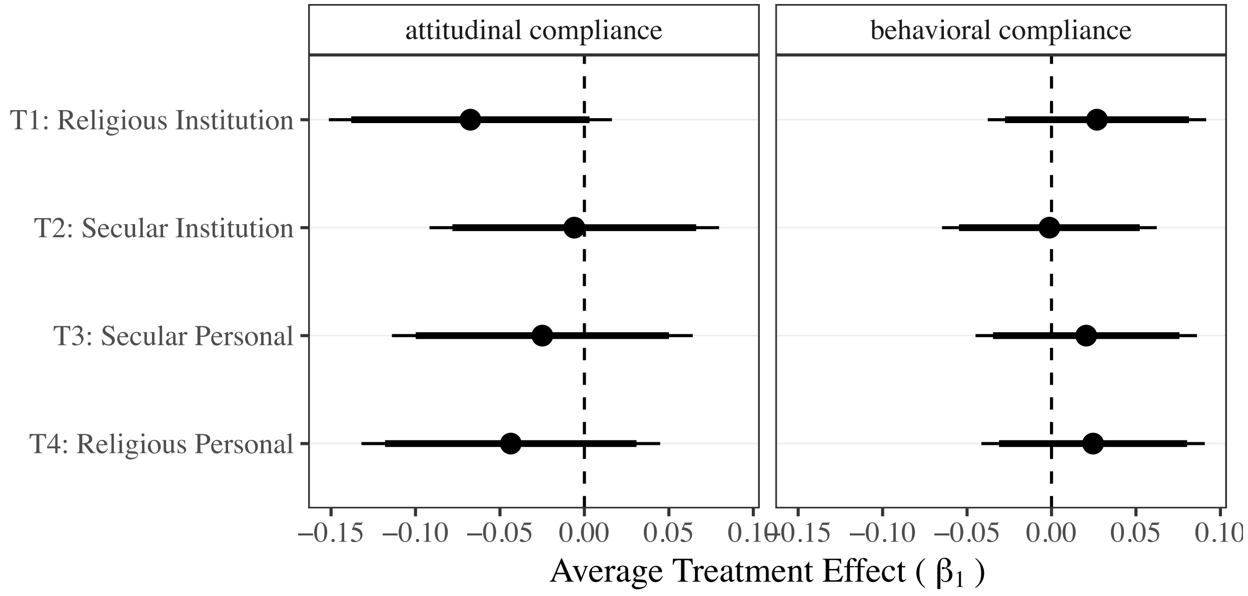

Figure 3—Individual Treatment Analysis, Hypotheses 8a-11b. Beta coefficients estimated from Equation 1. Conventional standard errors calculated at the individual level. 95% and 90% confidence intervals are included.
